# A variant of sparse partial least squares for variable selection and data exploration

**DOI:** 10.3389/fninf.2014.00018

**Published:** 2014-03-03

**Authors:** Megan J. Olson Hunt, Lisa Weissfeld, Robert M. Boudreau, Howard Aizenstein, Anne B. Newman, Eleanor M. Simonsick, Dane R. Van Domelen, Fridtjof Thomas, Kristine Yaffe, Caterina Rosano

**Affiliations:** ^1^Department of Biostatistics, University of PittsburghPittsburgh, PA, USA; ^2^Department of Epidemiology, Center for Aging and Population Health, University of PittsburghPittsburgh, PA, USA; ^3^Departments of Psychiatry, Bioengineering and Clinical and Translational Science, University of PittsburghPittsburgh, PA, USA; ^4^Department of Epidemiology, Graduate School of Public Health, University of PittsburghPittsburgh, PA, USA; ^5^Intramural Research Program, National Institute on AgingBaltimore, MD, USA; ^6^Department of Biostatistics, Emory UniversityAtlanta, GA, USA; ^7^Department of Preventive Medicine, University of Tennessee Health Science CenterMemphis, TN, USA; ^8^Department of Psychiatry, Neurology and Epidemiology, University of California, San FranciscoSan Francisco, CA, USA

**Keywords:** high-dimensional, multicollinearity, over-fitting, SPLS, inference, tuning parameters, network, MRI

## Abstract

When data are sparse and/or predictors multicollinear, current implementation of sparse partial least squares (SPLS) does not give estimates for non-selected predictors nor provide a measure of inference. In response, an approach termed “all-possible” SPLS is proposed, which fits a SPLS model for all tuning parameter values across a set grid. Noted is the percentage of time a given predictor is chosen, as well as the average non-zero parameter estimate. Using a “large” number of multicollinear predictors, simulation confirmed variables not associated with the outcome were least likely to be chosen as sparsity increased across the grid of tuning parameters, while the opposite was true for those strongly associated. Lastly, variables with a weak association were chosen more often than those with no association, but less often than those with a strong relationship to the outcome. Similarly, predictors most strongly related to the outcome had the largest average parameter estimate magnitude, followed by those with a weak relationship, followed by those with no relationship. Across two independent studies regarding the relationship between volumetric MRI measures and a cognitive test score, this method confirmed *a priori* hypotheses about which brain regions would be selected most often and have the largest average parameter estimates. In conclusion, the percentage of time a predictor is chosen is a useful measure for ordering the strength of the relationship between the independent and dependent variables, serving as a form of inference. The average parameter estimates give further insight regarding the direction and strength of association. As a result, all-possible SPLS gives more information than the dichotomous output of traditional SPLS, making it useful when undertaking data exploration and hypothesis generation for a large number of potential predictors.

## Introduction

In fields such as neuroscience, chemometrics, and genetics, data is often collected on a large number of variables but with a relatively small sample size, and predictors may also be highly collinear. Statistical methods used in this setting include regression models, cluster analysis and/or tree-based methods, ridge regression and dimension-reduction techniques such as partial least squares (PLS). However, when variable selection is the goal, these may prove inadequate or difficult to interpret.

In the realm of ordinary least squares (OLS), multicollinearity affects both the stability of the estimated coefficients (Wold et al., 1984) and inference on these estimates (Farrar and Glauber, 1967). Essentially, model prediction ability is poor when estimates are unstable (Wold et al., 1984), and one cannot trust conclusions drawn from test statistics, *p*-values or confidence intervals due to artificially inflated standard errors (Farrar and Glauber, 1967). As an alternative to OLS, ridge regression (Hoerl and Kennard, 2000; McDonald, 2009) and PLS account for multicollinearity and/or over-fitting. However, they are not intended for variable selection without additional computation such as bootstrapping (Abdi, 2010).

In PLS, latent variables (linear combinations of the predictors) are formed using both the outcome(s) and predictors such that all pairs of latent variables are orthogonal and have a sample correlation of zero (Garthwaite, 1994). Regression models are then fit using these latent variables rather than the original predictors and multicollinearity is no longer a concern. In addition, the number of latent variables is often smaller than the number of predictors, so that PLS reduces the dimensionality of the data and the likelihood of over-fitting. However, all predictors are assigned a non-zero weight and inference is not provided, so that variable selection is not readily achieved (Tobias, 1997; Chun and Keleş, 2010). Further detail on the theory underlying PLS regression is available elsewhere (Garthwaite, 1994; Wold et al., 2001; Krishnan et al., 2011).

Given standard PLS is not intended for variable selection but rather prediction, sparse methods such as sparse partial least squares (SPLS) were developed. Variable selection is accomplished by using tuning parameters in the modeling process, which drive both the latent variable selection and computation of predictors' weights (Chun and Keleş, 2010). Here, estimates may be set to zero, indicating a predictor is not significantly associated with the outcome.

Although some weights are zero so as to provide variable selection, this can also be viewed as a weakness of SPLS. In data exploration and hypothesis generation, effect size and *p*-values, despite insignificance, are often of interest. During exploratory analyses, one may wish to increase the type-I error rate and allow variables that would otherwise be borderline significant or insignificant into the set of selected predictors. Also, one may wish to compare standardized estimates of various predictors despite insignificance. None of this information is provided by executing SPLS in its traditional manner.

To address these shortcomings, an alternative approach, referred to here as “all-possible” SPLS, is proposed. Briefly, a SPLS model is fit for “all possible” values of the model's tuning parameters, as opposed to fitting only one model based on the “optimal” parameters (this latter approach will be referred to as “traditional” SPLS). Predictors are ranked by the percentage of time they are chosen across all models, and the average of non-zero standardized parameter estimates is given for all predictors, even those not chosen by traditional SPLS. Although not formal inference such as a *p-*value, the former gives the relative ranking of predictors, allowing one to identify potentially borderline significant variables, as well as those least likely to be predictive of the outcome. Simulation confirms predictors most strongly associated with the outcome are robust to changes in the tuning parameters and continue to be selected as sparsity increases, while those with the weakest association are less likely to be chosen under high levels of sparsity. This approach yields supplementary information lost in the traditional application of SPLS, providing increased insight into one's data.

## Methods

### Traditional SPLS

The spls package (version 2.1-0) in R (version 2.13.2) based on the theory presented by Chun and Keleş (2010) is considered here. The algorithm requires the specification of two tuning parameters, *K* and *η*. *K* (an integer between 1 and min{*p*, (*v* - 1)*n*/*v*}, where *v* is the number of folds for the cross-validation (CV), *p* is the number of predictors and *n* is the sample size (Chung et al., 2009)) is the number of latent variables and *η* (a continuous value on the interval [0, 1)) determines the amount of sparsity in the algorithm. In general, lower values of *η* represent less sparsity (and thus more variables tend to be selected), whereas higher values imply more sparsity. However, the choice of *K* also affects variable selection in conjunction with *η* (lower values of *K* tend to result in fewer chosen variables).

To facilitate the choice of *K* and *η*, the package includes a CV function, where the “optimal” *K* and *η* are those with the lowest mean squared prediction error. For the purposes of this paper, “traditional” SPLS refers to the use of this CV to choose one pair of “optimal” tuning parameters. Once determined, the SPLS model is fit and selected predictors are noted.

While using traditional SPLS, it was discovered the selection of optimal tuning parameters was affected by the seed if CV other than leave-one-out (LOO) was used. For example, for 1000 randomly-chosen seeds, the optimal values of the tuning parameters chosen most often by a 10-fold CV in the real data used in Section Data Application: Volumetric MRI Regions as Predictors of Cognitive Test Results were *K* = 2, *η* = 0.7. However, they were only chosen for 171 seeds out of 1000—about 17% of the time. The next pair chosen most often was *K* = 3, *η* = 0.6, at 106 times. All of the remaining pairings were chosen less than 10% of the time, so that no one pair was selected notably more than the others. Note that if *K* and/or *η* differ only by one unit, this can mean the addition or exclusion of one or more variables from the results. Here, eight predictors were chosen by the first set of tuning parameters, whereas 17 were chosen by the second, indicating instability in the tuning parameter values can cause instability in the variable selection process, affecting conclusions. Because of the unreliability of the 10-fold CV with these data, LOO CV is recommended for traditional SPLS.

Another consideration with the CV is how fine of a grid to use when searching for the optimal value of *η*, since, again, it is continuous. In the examples provided by the authors of the spls package, *η* may be one of 0.1, 0.2, 0.3, …, 0.9 (Chung et al., 2009; Chun and Keleş, 2010). Given this, and also the fact that considering more *η*-values results in significantly more computational time, *η*-values of 0.1, 0.2, 0.3, …, 0.9 were used in this paper as well.

### “All-possible” SPLS

“All-possible” is quoted because, given *η* is continuous, one cannot actually achieve every possible combination of tuning parameters. Given a discrete subset of *η* (here, {0.1, 0.2, …, 0.9}), however, one considers “all possible” combinations of the parameters. Specifically, there will be *K* × *η* total models fit, one for each combination of *K* and *η*, with standardized estimates recorded in each instance. The results are the percentage of time chosen (i.e., the parameter estimate was non-zero), as well as the average non-zero standardized parameter estimate.

It should be noted that with this method it is expected all predictors will be chosen a reasonable number of times (usually in at least 70% of the models). This is because once a large enough *K*- and/or small enough *η*-value is used, the method no longer induces enough sparsity to allow for variable selection—it essentially acts like PLS and chooses all variables. Since *all* pairings of *K* and *η* were considered here, many of them resulted in all variables being selected.

There are two advantages to all-possible SPLS. First, by ranking the variables based on how often they are chosen across all models, one has a relative way to compare them, as opposed to “chosen” or “not chosen.” Specifically, one can see those variables selected most and least frequently, as well as those that were somewhere in between. In this way, one obtains a continuum of information instead of a dichotomy. Second, an effect size for all predictors—not just those chosen by traditional SPLS—is provided. Thus, even if a predictor was only selected 75% of the time, one still has information on its estimate whenever it *was* selected.

## Simulation

### Simulation structure

A design analogous to that in Chun and Keleş (2010) was used to create collinear predictors of varying association with the outcome—one set of predictors was strongly associated, another weakly and a third not at all. For *j* = 1, 2, 3 and *c*_*j* − 1_ + 1 ≤ *i* ≤ *c_j_*, where (*c*_0_, *c*_1_, *c*_2_, *c*_3_) = (0, 7, 17, 27), predictors were of the form **x**_***i***_ **=** **m**_***j***_ + **ε**_***i***_. Given a sample size of *n* = 100, **m**_***j***_ were each vectors of length 100 from *N*(**0**, 20**I**_100_) and **ε**_***i***_ ~*N*(**0**, **I**_100_). Lastly, **y** = 2**m**_1_ − 0.2**m**_2_ + **τ**, where **τ** ~*N*(**0**, **I**_100_). All variables were standardized while other settings for the SPLS function were kept at default.

### Predictors with weaker association are less likely to be chosen with increased sparsity

This simulation demonstrated how predictors with varying levels of association with **y** are affected by changes in the tuning parameter pair, (*K*, *η* ). The general pattern is that for lower values of *K* and higher values of *η*, sparsity increases and fewer variables are selected. Here, *K* = {1, …, 27} and again *η* = {0.1, …, 0.9}.

**Figure 1:**
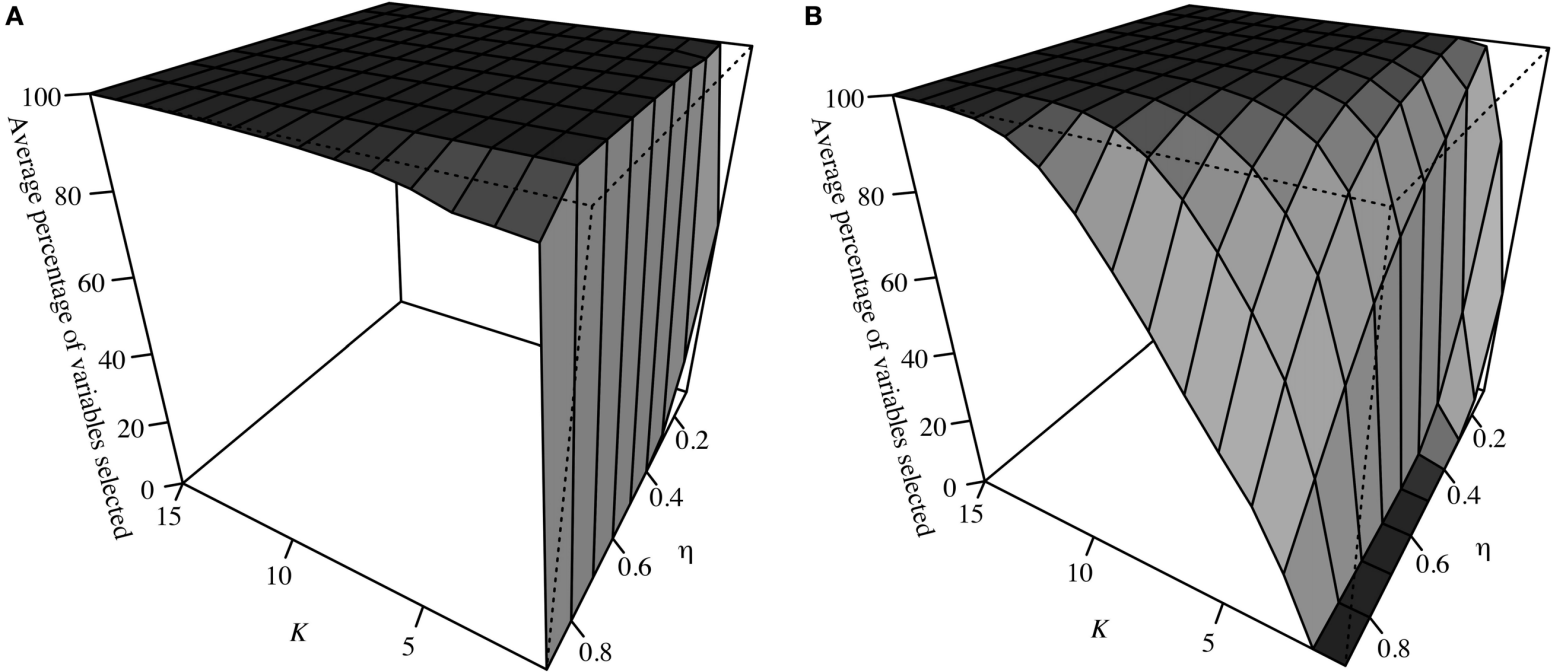
**(A)** shows the average percentage of variables in **S**_2_ selected for each pair of tuning parameters across *D* = 1000 simulated data sets, while **(B)** shows this for **S**_3_.

Consider three sets of predictors: **S**_1_ = {**x**_1_, …, **x**_7_} (strongly associated with **y**), **S**_2_ = {**x**_8_, …, **x**_17_} (weakly associated) and **S**_3_ = {**x**_18_, …, **x**_27_} (not associated). For each *d* = 1, …, *D* = 1000 samples drawn randomly from the distribution as outlined in Section Simulation Structure, a SPLS model was run for all pairs of *K* and *η*. The percentage of predictors chosen from each set was noted for each pair and the average across all 1000 data sets is shown in Figures 1A,B for **S**_2_ and **S**_3_. Note that *K* only ranges from 1 to 15, as after *K* = 15, the average was 100% for all pairs of tuning parameters. For **S**_1_, all seven predictors were always chosen (i.e., the average was always 100%).

These results confirm variables in set **S**_3_ (not associated with **y**) were less likely to be chosen as *K* decreased and *η* increased (i.e., sparsity increased). Variables in **S**_2_ showed a similar pattern due to their weak association, although their rate of selection was notably higher than those in **S**_3_. The fact that all variables in **S**_1_ were chosen for 100% of the (*K*, *η* ) pairs across all *D* data sets shows strongly associated variables are robust to changes in the tuning parameters. Subsequently, calculating the percentage of time a variable is selected over all pairs of tuning parameters (i.e., conducting all-possible SPLS) will result in those with the strongest association having the highest percentage of time chosen, while the opposite will be true for those with the weakest. This is shown via simulation in the next section.

### Percentage of time chosen and average non-zero standardized estimates

For each of *d* = 1, …, *D* = 1000 samples from the distribution as described in Section Simulation Structure, all-possible SPLS was conducted: For a given data set, a SPLS model was run for all pairs of *K* = {1, …, 27} and *η* = {0.1, …, 0.9}. Recorded was the percentage of time each variable was chosen, as well as the mean non-zero standardized parameter estimates. Table 1 reports the average of these percentages and mean estimates across all 1000 samples, in order to assess the method's behavior in the long run.

**Table 1:** **From all-possible SPLS conducted on *D* = 1000 samples from the same distribution**.

**Predictor**		**Average percentage of time chosen**	**Average of mean non-zero standardized $\hat{\beta }$**
**S**_1_	**x**_1_	100	0.144
	**x**_2_	100	0.147
	**x**_3_	100	0.145
	**x**_4_	100	0.142
	**x**_5_	100	0.144
	**x**_6_	100	0.147
	**x**_7_	100	0.145
**S**_2_	**x**_12_	96.5	−0.011
	**x**_11_	96.482	−0.011
	**x**_17_	96.476	−0.010
	**x**_8_	96.474	−0.011
	**x**_10_	96.472	−0.011
	**x**_15_	96.466	−0.012
	**x**_16_	96.465	−0.010
	**x**_14_	96.460	−0.007
	**x**_14_	96.453	−0.009
	**x**_9_	96.451	−0.011
**S**_3_	**x**_20_	89.912	0.0011
	**x**_21_	89.886	−0.0003
	**x**_24_	89.851	−0.0051
	**x**_18_	89.839	−0.0026
	**x**_27_	89.786	0.0021
	**x**_23_	89.778	0.0038
	**x**_26_	89.770	0.0007
	**x**_22_	89.734	−0.0023
	**x**_25_	89.730	0.0001
	**x**_19_	89.710	0.0035

Average percentage of time a variable was chosen and its average mean non-zero standardized parameter estimate across D data sets. Variables are ordered by average percentage.

The average percentage of time chosen for all predictors in **S**_1_ was 100, while those in **S**_2_ and **S**_3_ were all chosen around 96% and 90% of the time on average, respectively, resulting in three distinct groups. The average mean non-zero standardized estimates for those in **S**_1_ were all around 0.15, while those in **S**_2_ were about −0.01, and those in **S**_3_ were always smaller than those in **S**_2_ (and **S**_1_). Both the magnitudes and directions of the estimates for **S**_1_ and **S**_2_ were as expected given the structure of the data outlined in Section Simulation Structure and the fact that estimates were standardized. The small magnitudes and varying directions of predictors in **S**_3_ were reasonable, as they should have estimates that hover around zero.

## Data application: volumetric MRI regions as predictors of cognitive test results

In neuroimaging, brain regions tend to be numerous and highly correlated, so that over-fitting and multicollinearity are of concern. Here, a well-established predictor-outcome relationship is used to illustrate the proposed SPLS method.

### Data collection

#### Participants

Data were obtained from the Cardiovascular Health Study (CHS), which is an ongoing, population-based, longitudinal study, and the Healthy Brain Project (HBP), a sub-study of the Health, Aging and Body Composition (Health ABC) Study, which is also longitudinal and population-based.

The CHS is a study of coronary heart disease and stroke risk in older adults. Briefly, 5888 community-dwelling older adults were identified between 1987 and 1993 from Medicare eligibility lists in four clinical centers (Forsyth County, NC; Sacramento County, CA; Washington County, MD and Pittsburgh, PA) (Fried and Borhani, 1991). Participants were recruited if they were age 65 or older at time of recruitment, non-institutionalized, not wheelchair-bound or undergoing active cancer treatment, able to give informed consent and expected to remain in the area for at least 3 years. The participants had annual clinic examinations through 1998–1999.

Brain MRIs were acquired for 523 participants in Pittsburgh in 1997–1999 (Lopez et al., 2003). Compared to the participants who did not have a brain MRI, these participants were younger, more likely to have more years of education and had a lower prevalence of cardiovascular diseases and cerebrovascular findings (Rosano et al., 2006, 2007a). In 2003–2004, a random sample of 327 brain MRIs from the 523 were re-read (Rosano et al., 2005, 2007a,b, 2008). No significant differences were observed with regard to demographics or health-related factors between these 327 participants and the 523 total subjects.

The Health ABC study began in 1997–1998 as a longitudinal, observational cohort study of 3075 well-functioning older adults from Pittsburgh, PA and Memphis, TN (Simonsick et al., 2001). Participants were enrolled if they were 70–79 years old and reported no difficulty walking a quarter of a mile (400 meters), climbing 10 steps or performing activities of daily living; were free of life-threatening cancers with no active treatment within the prior 3 years and had planned to remain within the study area for at least 3 years. In 2006–2007, 314 Health ABC participants from the Pittsburgh site who were interested in and eligible for a brain 3T MRI received a MRI in addition to in-person Health ABC assessments. This ancillary study of the Health ABC is referred to as the HBP.

Both studies have been approved by the institutional review boards of the University of Pittsburgh.

#### Magnetic resonance imaging (MRI) measures

In both the CHS and HBP, brain MRI assessments included volumetric measures of gray matter for both individual regions and the whole brain.

The brain MRI protocol for the CHS carried out in 1997–1999 has been described elsewhere (Yue et al., 1997). Briefly, sagittal T1-weighted localizer sequences and axial spin-echo spin-density-weighted, spin-echo T2-weighted and T1-weighted images were acquired using a 1.5T scanner. A volumetric Spoiled Gradient Recalled Acquisition (SPGR) sequence with parameters optimized for maximal contrast among gray matter, white matter and cerebrospinal fluid (CSF) was acquired in the coronal plane (echo time/repetition time (*TE*/*TR*) = 5/25, flip angle = 40 deg., NEX = 1, slice thickness = 1.5/0 mm interslice). All MRI data were interpreted at a central MRI Reading Center using a standardized protocol (Bryan et al., 1997; Yue et al., 1997).

The protocol for the HBP study was performed with a Siemens 12-channel head coil and 3T Siemens Tim Trio MR scanner at the Magnetic Resonance Research Center, University of Pittsburgh (Venkatraman et al., 2011; Rosano et al., 2012a,b). Magnetization-prepared rapid gradient echo T1-weighted images (MPRAGE) were acquired in the axial plane (*TR* = 2300 ms, *TE* = 3.43 ms, imaging time (*TI*) = 900 ms, 9° flip angle, 256 × 224 mm field of view (FOV), 1 × 1 mm voxel size, 256 × 224 matrix size, 176 slices and 1 mm thick). Fluid-attenuated inversion recovery (FLAIR) images were acquired in axial plane (*TR* = 9160 ms, *TE* = 89 ms, *TI* = 2500 ms, 150° flip angle, 256 × 212 mm FOV, 256 × 240 matrix size, 48 slices, 3 mm thick and 1 × 1 mm voxel size). Diffusion-weighted images were acquired using a single short spin-echo sequence (*TR* = 5300 ms, *TE* = 88 ms, *TI* = 2500 ms, 90° flip angle, 256 × 256 mm FOV, two diffusion values of *b* = 0 and 1000 s/mm, 12 diffusion directions, four repetitions, 40 slices, 3 mm thick, 128 × 128 matrix size, 2 × 2 mm voxel size and GRAPPA = 2). A neuroradiologist examined each MRI for neurologic abnormalities. A radiologist verified the presence of abnormalities with potential clinical relevance. No images were excluded because of unexpected findings.

Voxel counts of the gray matter were obtained for individual regions of interest and for the whole brain using a procedure previously described (Zhang et al., 2001; Tzourio-Mazoyer et al., 2002; Rosano et al., 2005; Wu et al., 2006). After skull and scalp stripping (Smith, 2002), and after segmentation of gray matter, white matter and CSF, the brain atlas and the individual subject brain were aligned and intensity normalization was done on each subject's structural image (SPGR for the CHS and MPRAGE for the HBP images), as well as on the template colin27, to give each subject the same orientation and image intensity distribution as the template and to improve the registration accuracy. For both the CHS and HBP, FMRIB-FAST was applied to segment the image into gray matter, white matter and CSF, while also correcting for spatial intensity variations such as bias field or radio-frequency inhomogeneities (Rosano et al., 2005; Wu et al., 2006). The registration procedure used a fully-deformable automatic algorithm (Thirion, 1998) that does not warp or stretch the individual brain, and thus minimizes measurement inaccuracies (Wu et al., 2006). Volumes were converted from number of voxels to cubic millimeters.

### Data description and preparation

#### Dependent variable

Scores from the Modified Mini-Mental State Examination (3MS) were used as the dependent variable, as it is a highly studied outcome with regard to memory. The 3MS is a brief, general cognitive battery with components for orientation, concentration, language, praxis and immediate and delayed memory (Teng and Chui, 1987). Because scores tend to be clustered at the high end of the scale, a transformation for left-skewed data was used:-ln(101 - 3*MS*), where 3*MS* represents the test score for a given individual (Shackman et al., 2006).

#### Regions of interest and confounding variables

A tiered hypothesis was formed based on the strength of current findings, with the expectation that primary regions would have the strongest association with 3MS, followed by secondary regions. A third set of regions referred to as “non-hypothesized” were not expected to be associated with the outcome.

The primary hypothesized regions were the hippocampus, parahippocampus and entorhinal cortex (Zola-Morgan and Squire, 1993; Dickerson et al., 2001). The secondary hypothesis included additional memory-related regions: amygdala, caudate and medial parietal, lateral parietal and posterior cingulate cortices (Packard and Knowlton, 2002; Koivunen et al., 2011; Squire and Wixted, 2011). Lastly, non-hypothesized regions were those traditionally related to motor tasks and performance (not memory): putamen, pallidum, thalamus, supplementary motor cortex, cerebellum, and post-central and pre-central gyri (Rosano et al., 2007a). Because the pallidum measurements were highly skewed right, the natural logarithm of these values was used. Regions were not normalized, as total gray matter parenchyma was included as a covariate.

The following variables were included as predictors in all models because of prior work indicating an association with 3MS and/or brain structure (Brickman et al., 2008; Raji et al., 2010): race (coded as white and all other races), sex, age, obesity (indicated by a BMI greater than 30) and total brain parenchyma volume (here, represented by total gray matter volume). The treatment of confounding variables here is analogous to that in the OLS regression framework: They were included in all models and never removed, even if they were ultimately not significant. Thus, the interpretation of a set of selected variables is that they are significantly related to the outcome, controlling for confounding variables and all other brain regions.

#### Influential points

Before the analysis commenced, potentially influential data points were determined by modeling each predictor against each outcome individually and calculating externally studentized residuals in each case (SAS Institute Inc, 2008). Any observation with a residual greater than 2.5 in absolute value was removed from the analysis (this value is slightly less conservative than the cut-off of 2 suggested by the SAS documentation).

Three observations were removed from the HBP data based on the above criterion, while 11 were removed from the CHS. In both data sets, influential points were those with a notably small/large 3MS value paired with a large/small regional volume. The only exception was one observation in the HBP data, which had a very large total brain volume relative to the other subjects. For each data set, there were some subjects with invalid MRIs and/or missing covariate values, so that after removing these subjects and also the influential observations, the final sample size for the CHS was *n* = 286, while *n* = 302 for the HBP. In Table 2, *p*-values for differences in demographic measures between the CHS and HBP cohorts were obtained either by a chi-square test, two-sample *t*-test or the Kruskal-Wallis Test when normality was suspect.

**Table 2:** **Demographic and MRI volumetric summaries for Cardiovascular Health Study and Healthy Brain Project participants**.

	**Cardiovascular health study**	**Healthy brain project**	***p*-value**
Sample size	*n* = 286	*n* = 302	
Female (n, %)	177 (62%)	174 (58%)	0.33
White (n, %)	224 (78%)	181 (60%)	<0.001^a^
Obese (n, %)	46 (16%)	79 (26%)	0.004^a^
Age [mean (*SD*)]	78 (4.0)	83 (2.8)	<0.001^a^
3MS score [mean (*SD*)]	93.6 (5.2)	92.9 (6.7)	0.92
MRI volumes [mean mm^3^ (*SD*)]^b^			
Amygdala	2786 (605)	2934 (419)	
Anterior cingulate cortex	10554 (1587)	9615 (1517)	
Caudate	7704 (1835)	8586 (2047)	
Cerebellum	68220 (24799)	99264 (12788)	
Dorsolateral prefrontal cortex	67790 (9706)	26837 (3240)	
Entorhinal cortex	3922 (808)	3702 (664)	
Hippocampus	9649 (1296)	9452 (1205)	
Lateral parietal inferior cortex	10368 (2061)	10990 (1527)	
Lateral parietal superior cortex	9719 (2342)	11787 (1807)	
Medial parietal cortex	18483 (3408)	20611 (3031)	
Pallidum (natural logarithm)	5.49 (1.11)	5.90 (0.93)	
Parahippocampus	10144 (1570)	10663 (1608)	
Parenchyma (total gray matter)	466482 (66738)	527997 (55062)	
Post-central gyrus	15255 (3132)	18972 (2730)	
Posterior cingulate cortex	2557 (458)	2816 (695)	
Pre-central gyrus	13485 (2579)	16741 (2538)	
Putamen	2192 (1946)	3002 (2443)	
Supplementary motor cortex	9260 (2168)	128218 (2220)	
Thalamus	1872 (1016)	1856 (460)	

a Significant with α = 0.05.

b Mean volumes are expected to differ since the CHS and HBP used different MR scanners, thus p-values are not reported.

Analyses were conducted using R version 2.13.2 (spls package 2.1-0) and SAS version 9.2 (SAS Institute Inc, 2008). Both the dependent and continuous independent variables were standardized, and, unless otherwise mentioned, all other settings were kept at default for all functions/procedures used. Run-time for the SPLS analyses of interest was less than 5 minutes on a machine with the Windows 7 operating system (64 bit) and a 2.16 GHz Intel Core i7 processor.

### Multicollinearity and over-fitting

Multicollinearity was assessed using the condition number by fitting an OLS regression model that included all regions of interest and *a priori* confounders, where a value greater than 100 indicated significant multicollinearity (Belsley et al., 1980).

The CHS cohort had a condition number of 190, while the HBP group had a value of 227. Since both are notably larger than 100, multicollinearity is likely present in these data when all MRI regions are considered simultaneously in the same model (Belsley et al., 1980).

While the number of predictors (23) was not larger than the sample sizes (297 and 302), various rules of thumb indicate there should be 10–20 observations for each predictor in a model (Harrell, 2001). This suggests one should have at least 230 observations, and potentially as many as 460, which could indicate potential over-fitting with these data.

### Sparse partial least squares analysis

The spls package based on the theory presented by Chun and Keleş (2010) was used for both traditional and all-possible SPLS (Tables 3, 4). Horizontal lines show potential empirically-driven cut-points that indicate varying levels of association between the predictors and outcome.

**Table 3:** **Results from all-possible (first two columns) and traditional (last column) SPLS for the Healthy Brain Project**.

**Brain region**	**% Times chosen with all-possible method**	**Mean non-zero $\hat{\beta }$ from all-possible method**	**Traditional SPLS $\hat{\beta }$^c^**
^a^Hippocampus	100	0.276	0.262
^a^Parahippocampus	100	0.258	0.180
^b^Amygdala	97.6	0.137	0.065
Anterior cingulate cortex	96.6	0.088	0.091
^a^Entorhinal cortex	96.1	−0.279	−0.159
^b^Medial Parietal cortex	96.1	0.148	0.158
Supplementary motor cortex	96.1	−0.187	−0.310
Thalamus	95.2	−0.104	−0.098
Dorsolateral prefrontal cortex	92.3	0.071	0.020
^b^Lateral parietal superior cortex	91.8	−0.163	−0.101
Pallidum (natural logarithm)	90.3	−0.106	−0.113
^b^Lateral parietal inferior cortex	89.4	0.068	
Post-central gyrus	88.4	0.056	0.079
Putamen	85.0	0.031	
Pre-central gyrus	84.5	−0.024	
Cerebellum	84.1	0.006	0.012
^b^Caudate	83.6	−0.021	
^b^Posterior cingulate cortex	82.6	−0.033	

Regions are ordered by the percentage of time chosen across all models by the all-possible method (first column), with the horizontal lines separating potential groupings of predictors with regard to their strength of association with the outcome.

a Primary hypothesized region.

b Secondary hypothesized region.

c Applicable only for those regions chosen by traditional SPLS using optimal tuning parameters.

**Table 4:** **Results from all-possible (first two columns) and traditional (last column) SPLS for the Cardiovascular Health Study**.

**Brain region**	**% Times chosen with all-possible method**	**Average non-zero $\hat{\beta }$ from all-possible method**	**Traditional SPLS $\hat{\beta }$^c^**
^a^Parahippocampus	100	0.196	0.111
^a^Hippocampus	100	0.141	0.093
^b^Medial parietal cortex	100	0.228	0.054
^b^Lateral parietal inferior cortex	97.6	0.220	0.060
Pallidum (natural logarithm)	96.6	0.131	0.126
Pre-central gyrus	96.1	0.196	0.021
^b^Lateral parietal superior cortex	94.7	−0.290	
Dorsolateral prefrontal cortex	92.8	0.013	0.026
Supplementary motor cortex	90.8	−0.128	−0.110
^b^Amygdala	89.9	0.053	0.084
^a^Entorhinal cortex	89.4	−0.132	
^b^Posterior cingulate Cortex	88.9	−0.062	
Thalamus	88.9	−0.078	
Post-central gyrus	87.0	−0.070	
Putamen	84.1	0.054	
Anterior cingulate cortex	82.6	0.005	
^b^Caudate	79.7	0.022	
Cerebellum	79.2	0.050	

Regions are ordered by the percentage of time chosen across all models by the all-possible method (first column), with the horizontal lines separating potential groupings of predictors with regard to their strength of association with the outcome.

a Primary hypothesized region.

b Secondary hypothesized region.

c Applicable only for those regions chosen by traditional SPLS using optimal tuning parameters.

Within the HBP data set (Table 3), all-possible SPLS largely confirmed the proposed hypotheses by choosing two of the primary regions (hippocampus and parahippocampus) 100% of the time and the third (entorhinal cortex) in 96.1% of the models. Additionally, the three largest average non-zero parameter estimates from all-possible (second column) were for the three primary regions: entorhinal cortex (−0.279), hippocampus (0.276) and parahippocampus (0.258). This contrasts traditional SPLS in that the region with the largest estimated magnitude (third column) was the supplementary motor cortex (−0.310), yet this was not a hypothesized region. Although chosen a relatively large percentage of the time by all-possible (96.1%), this region was ranked below/tied with all three primary regions and two secondary (amygdala, medial parietal cortex). It also had a smaller average estimate (−0.187) than all three primary regions. Thus, this region was deemed most significant by traditional, but ranked below multiple hypothesized regions by all-possible.

Traditional SPLS also chose post-central gyrus and cerebellum, so that one might conclude these regions are significantly predictive of 3MS, yet cerebellum was the third lowest-ranked region by all-possible (84.1%), and post-central the sixth lowest (88.4%).

Lastly, the additional information gained by all-possible SPLS (ranking according to percent) indicates the lateral parietal inferior cortex is a potentially borderline significant predictor (89.4%), which could not have been known based on the traditional results, as its parameter estimate was set to zero.

Despite being secondary regions, neither the caudate nor posterior cingulate cortex were chosen by either method, so that the results were consistent in this way and may indicate a different relationship in a multivariable setting than has been seen in previous studies involving individual predictors.

The CHS results (Table 4) are notably consistent with those from the HBP data. Specifically, two primary regions (parahippocampus, hippocampus) were again chosen in 100% of the models, although the third primary region (entorhinal cortex) was selected less often, at 89.4%. However, this region had a larger average parameter estimate (−0.132) than all other regions selected less than 90% of the time, and some regions selected in greater than 90% of the models (pallidum, dorsolateral prefrontal and supplementary motor cortices, all non-hypothesized). This again shows the utility of all-possible SPLS in that it highlighted a potentially important, borderline predictor that was missed by traditional.

The regions with the largest average magnitudes according to all-possible were the lateral parietal superior (−0.290), medial parietal (0.228) and lateral parietal inferior (0.220) cortices (all secondary), and the parahippocampus (0.196), a primary region, so that the top four largest estimates were associated with hypothesized regions. Alternatively, traditional SPLS assigned the largest parameter estimate to the pallidum (0.126), followed by the parahippocampus (0.111) and the supplementary motor cortex (−0.110), so that two of the three regions with the largest estimates according to traditional SPLS were non-hypothesized. In contrast, all-possible ranked both the pallidum (96.6%) and supplementary motor (90.8%) lower than two primary (parahippocampus, hippocampus) and two secondary (medial parietal, lateral parietal inferior cortices) regions (and also lower than lateral parietal superior in the case of the supplementary motor cortex).

Lastly, the posterior cingulate cortex and caudate, despite being secondary regions, were not chosen by either method. This finding for the caudate is consistent with that from the HBP.

## Discussion

The purpose of this study was to illustrate that all-possible SPLS provides additional, useful information not attainable by traditional SPLS: relative rankings and parameter estimates for non-selected predictors. Simulation verified that predictors not associated with the outcome are selected less often as sparsity increases, while strong, and in most cases weak, associations remain robust. Additionally, conducting all-possible SPLS a large number of times showed that, on average, the percentage of time chosen and mean non-zero standardized estimates were consistent with the structure of the simulated data. A real data example indicated all-possible SPLS was more successful at highlighting hypothesized relationships than traditional SPLS, and also gave useful information about borderline variables that could not otherwise have been known.

Given the CHS and HBP data sets differed with respect to neuroimaging protocols and demographics, it is notable that all-possible SPLS detected hypothesized associations across these cohorts, suggesting robustness in the method. Specifically, MR scanners had different field strengths: the CHS MRIs were obtained with a 1.5 Tesla, the HBP with a 3.0. Additionally, protocols with different spatial resolutions were used across groups: the CHS applied a 5.0 mm slice, whereas the HBP applied a 1.5. Lastly, the cohorts were significantly different with regard to race, obesity and age (although these factors were controlled for in all models). Despite these differences between data sets, the method yielded consistent results overall, indicating its utility as variable selection technique.

A weakness of all-possible SPLS is its relative nature (i.e., ranking by percentage) with no strict cut-off value due to a lack of distributional properties. For example, in the simulation in Section Percentage of Time Chosen and Average Non-Zero Standardized Estimates (Table 1), the average percentage defined three distinct groups, but with no insight into significance (or lack thereof). However, viewing the predictors in this way allows one to see more detail than the dichotomous results of traditional SPLS, and to apply a cut-off if desired, where the value would be based on empirical experience, rather than guided by theory.

By utilizing simulation and a well-studied predictor-outcome relationship across two independent studies, the current findings validate this variation of SPLS as a useful technique for selecting variables in situations where other approaches (namely, OLS) fail. The results of this study suggest all-possible SPLS could be used for hypothesis generation without having to restrict the set of predictors due to multicollinearity or a comparatively small sample size, which geneticists, neuroscientists, economists and social scientists often encounter. The additional information given by all-possible SPLS is especially useful in exploratory analyses, as it allows for a more thorough understanding of the data than can be provided by the binary results of traditional SPLS.

### Conflict of interest statement

The authors declare that the research was conducted in the absence of any commercial or financial relationships that could be construed as a potential conflict of interest.
